# Editorial: Advances in Early Onset Epilepsies

**DOI:** 10.3389/fneur.2021.709213

**Published:** 2021-07-15

**Authors:** Alasdair Patrick John Parker, Hans Hartmann, Patrick Van Bogaert

**Affiliations:** ^1^University of Cambridge, Cambridge, United Kingdom; ^2^Hannover Medical School, Hannover, Germany; ^3^Centre Hospitalier Universitaire d'Angers, Angers, Pays de la Loire, France

**Keywords:** epilepsy, genomics, neurometabolic, MRI, treatment

Early-onset epilepsies (EOE) elicited submissions reflecting the epidemiology, investigation, and management of these challenging epilepsies: key areas where there has been a significant upshift in investigation, including genomic, radiological, and electrophysiological technological advances and targeted therapies.

The genomic investigation of infantile and childhood epileptic encephalopathies has been transformed initially by gene panels and more recently by whole genome sequencing of both parents and child (trio). The power of this technology is demonstrated in the paper submitted from Kazakhstan—the first publication on genomic investigations in children with epilepsies from Central Asia.

Jaxybayeva et al. described the incidence and prevalence of early-onset epilepsies in their country. They identified children where gene panel investigation was undertaken and noted that in up to 80% the result was transformational. On a less positive note, they highlight genomic sequencing was unavailable to them within Central Asia, and sample analysis abroad meant they were unable to control the quality/accuracy of the reports. Their call for action within this area is timely and supported by their data.

Conversely, the Kenyan series of neuroimaging in children with epilepsy highlights the possibilities within both resource-rich and resource-poor countries. Samia et al. highlight that up to one-third of the children who underwent an MRI had a positive yield for abnormal findings. Their cohort was in contradistinction to the etiology of other studies performed in similar settings, with relatively few cases of infectious etiology. They cross-correlated with comorbidity/epileptiform activity and suggested appropriate prioritization/ranking for children undergoing further investigation.

The role of neuromonitoring in neonatal-onset epileptic encephalopathy is a reflection of practice within well-resourced health environments. Trollmann emphasizes the use of both standard/amplitude-integrated EEG in monitoring and etiological identification of rare genetic syndrome.

Taken together, these papers tell us that the investigation of EOE needs to be multi-axial, using up-to-date technology to allow for accurate classification and hence targeted therapy. This can be achieved in both resource-poor and resource-rich countries.

The epidemiology of epilepsies is well-covered by two papers covering both rare epilepsies and also a large national cohort.

Stülpnagel et al. present a very large series of children identified through the network for therapy and rare epilepsies (NETRE). The power of larger-scale population capture is well-demonstrated both within the identification of relatively new epilepsy syndromes, e.g., febrile infection-related epilepsy syndrome (FIRES), classification of different genomic variants, e.g., SCN2A, and both clinical features in certain epilepsy syndromes as well as their investigation findings giving a hallmark of the underlying etiology, e.g., the MRI findings in FOXG1.

Gibaud et al. synchronized with the NETRE study showing that studying large cohorts, in this case data from a French cohort/review of the literature on pyridoxine/pyridoxal phosphate-dependant epilepsies, concluding that their consideration is not needed in *de novo* West Syndrome (with no other signs or suggestive history). Their data is highly informative and is likely to simplify the therapeutic journey for these children from within France and further afield.

Refractory status epilepticus is a relatively rare occurrence, but often devastating. The papers by Schoeler et al. and Breu et al. highlight the great challenges but also potential solutions, including the early introduction of the ketogenic diet. The papers also highlight the difficulty in extrapolating data from retrospective case series. Controlled trials of interventions in very rare conditions in the ICU are very difficult to set up, but networks of care, such as NETRE, are a possible solution.

The paper by Assenza et al. targets symptom-specific therapy as opposed to the syndrome. Their case series and systematic review cover the role of a newer antiepileptic drug in cortical myoclonus, and they produce an evidence base for therapy of what are normally extremely challenging epilepsies.

However, many of the epilepsies described, despite benefiting from accurate classification and disease-specific therapy, will continue to be drug-resistant and require a high level of support in all societies.

Taken together, it is a very exciting time in the field of EOE. These papers reflect the enormous advances in most settings as well as the challenges that face us still.

## Author Contributions

AP initiated the process with the editorial writing the first draft. All authors contributed to the article and approved the submitted version.

## Conflict of Interest

The authors declare that the research was conducted in the absence of any commercial or financial relationships that could be construed as a potential conflict of interest.

